# Pharmacokinetics and Neuroprotective Efficacy of Poly‐Arginine Peptide R18D in Sprague Dawley Rats Subjected to Transient Intraluminal Filament Middle Cerebral Artery Occlusion Stroke

**DOI:** 10.1002/cns.70969

**Published:** 2026-06-03

**Authors:** Bruno P. Meloni, Stuart K. Gribble, Meghan G. Thomas, Ayelet Weksler, Lena Finkelstein, Lena Fishtein, Dimitry Kovalchuk, Gilad Maximov, Avital Schauder, Keren Kigel‐Zur, Sigal Meilin

**Affiliations:** ^1^ Perron Institute for Neurological and Translational Science Nedlands Western Australia Australia; ^2^ Department of Neurosurgery Sir Charles Gairdner Hospital, QEII Medical Centre Nedlands Western Australia Australia; ^3^ Centre for Neuromuscular and Neurological Disorders, the University of Western Australia Nedlands Western Australia Australia; ^4^ Argenica Therapeutics Nedlands Western Australia Australia; ^5^ MD Biosciences Innovalora, Ltd. Rehovot Israel

**Keywords:** MCAO, neuroprotection, poly‐arginine peptide, R18D, stroke

## Abstract

**Background:**

This study examined the dose‐dependent efficacy and pharmacokinetics of R18D in a rat transient middle cerebral artery occlusion (MCAO) stroke model.

**Methods:**

R18D was intravenously administered 60 min after the onset of a 90‐min MCAO induced by intraluminal filament insertion. For pharmacokinetic profiling, blood samples were collected 10, 15, 30 and 60 min, and at 2, 4 and 8 h after the start of a 10‐min intravenous infusion of R18D. Infarct volume and body weight were assessed at 24 h after MCAO.

**Results:**

R18D reduced mean total infarct volume by 47.2% (*p* = 0.0003), 28.4% (*p* = 0.008), and 27.9% (*p* = 0.003) at doses of 325, 650 and 1100 nmol/kg, respectively. The 650 nmol/kg dose significantly reduced cerebral hemisphere swelling by 8.3% (*p* = 0.01), whereas the 325 nmol/kg (5.6%; *p* = 0.15) and 1100 nmol/kg (4.5%; *p* = 0.14) doses produced non‐significant reductions in swelling. R18D did not significantly affect post‐stroke weight loss, however rats treated with the lowest dose (325 nmol/kg) exhibited the smallest reduction in body weight compared with vehicle‐treated animals (7.35% vs. 9.35). Pharmacokinetic analysis of the three R18D doses after stroke demonstrated a half‐life of 72 to 78 min, with dose‐dependent plasma Cmax values of 5.3 μg/mL, 11.9 μg/mL and 17.3 μg/mL for the low‐, medium‐ and high‐dose groups, respectively.

**Conclusions:**

These results further support the neuroprotective properties of R18D and provide strong justification for its continued development as a neuroprotective treatment for ischemic stroke.

## Graphical abstract

The R18D peptide exhibits a favorable pharmacokinetic profile and significantly reduces infarct volume in a rat model of middle cerebral artery occlusion (MCAO) followed by reperfusion. These findings highlight the potential clinical utility of R18D as a neuroprotective therapy for stroke patients undergoing endovascular recanalization interventions.

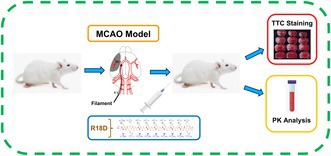

## 1 Introduction

Despite extensive experimental research and numerous clinical trials conducted over several decades, the development of an effective and safe neuroprotective therapy to minimize brain tissue damage remains an urgent unmet need and a major priority in ischemic stroke. Furthermore, the use of reperfusion therapies, including intravascular thrombolysis and, more recently, endovascular thrombectomy, highlights the need for an early neuroprotective therapy that can be administered prior to hospital admission. Such an approach could help preserve salvageable penumbral tissue and extend the therapeutic time window for recanalization interventions, thereby improving patient outcomes [1]. To this end, a neuroprotective agent capable of targeting multiple neurodamaging processes and/or stimulating multiple cell survival pathways is likely to offer the greatest potential for achieving neuroprotection in ischemic stroke.

R18D (known as ARG‐007 in clinical studies) is a synthetically derived poly‐arginine peptide comprising 18 positively charged D‐arginine residues and belongs to a novel class of cytoprotective compounds known as cationic arginine‐rich peptides (CARPs; reviewed in [2]). Numerous pre‐clinical studies have demonstrated that CARPs possess multiple cytoprotective properties [2], with extensive pre‐clinical evidence showing that R18D and its L‐enantiomer, R18, reduce brain injury following ischemic stroke [2, 3, 4, 5, 6, 7, 8, 9], hypoxic–ischemic encephalopathy (HIE) [10, 11] and traumatic brain injury (TBI) [12, 13, 14, 15] and excitotoxic neuronal death in vitro [7, 10, 16, 17].

Both R18D and R18 protect neurons from excitotoxicity by limiting intracellular calcium influx [10]. In addition, these peptides exert neuroprotective effects by multiple mechanisms, including mitochondria targeting and stabilization [18], inhibiting proteolytic enzymes such as matrix metalloproteinases (MMPs) [19], and reducing neuroinflammation [13]. Importantly, the D‐enantiomer structure of R18D confers resistance to degradation by tissue plasminogen activator (tPA) and plasmin during therapeutic thrombolysis [7, 17]. Furthermore, neither R18D nor R18 exacerbates ongoing bleeding following induced intracerebral hemorrhage in rats [20]. Due to its resistance to degradation during thrombolysis [7, 17], R18D was selected as the lead peptide for clinical development as a neuroprotective treatment for acute ischaemic stroke.

With respect to clinical development, a first‐in‐human study in healthy volunteers (R18D, *n* = 6 per dose; Placebo, *n* = 6) demonstrated that single intravenous doses of R18D at 0.03 mg/kg (10 nmol/kg), 0.1 mg/kg (33 nmol/kg), 0.2 mg/kg (67 nmol/kg) and 0.3 mg/kg (100 nmol/kg) were safe and well tolerated, with no dose related adverse effects observed (unpublished observation). In addition, in a Phase 2 clinical acute ischemic stroke study (SEANCON; R18D, *n* = 46; Placebo, *n* = 46. Australian New Zealand Clinical Trials Registry No.: ACTRN12623001110673), administration of a single intravenous dose of R18D at 100 nmol/kg within 24 h of symptom onset was also shown to be safe, and was associated with significantly smaller final infarct volumes in patients with larger baseline infarct cores (unpublished observation).

The R18 L‐enantiomer has previously been extensively investigated in experimental stroke models and has been shown to reduce infarct volume and/or improve functional outcomes (e.g., adhesive tape removal test, forelimb movement, non‐human primate stroke scale) in both permanent and transient middle MCAO stroke models induced using a intraluminal filament, clip or endothelin‐1 (ET‐1) [3, 4, 5, 6, 7, 8, 9, 21]. Similarly, R18D has also been demonstrated to reduce infarct volume in filament induced permanent MCAO models [6, 7] and to improve functional outcomes following transient ET‐1‐induced MCAO [8]. However, further investigation is required to identify optimal dosing regimens, and drug pharmacokinetics.

Therefore, this study aimed to evaluate the dose–response efficacy of the R18D enantiomer in reducing infarct volume and brain swelling, and to characterize its pharmacokinetic profile in an intraluminal filament‐induced transient MCAO model, which more closely resembles human thromboembolic strokes treated with recanalization interventions. Importantly, this study was conducted in an independent preclinical contract research organization, separate from previously published experiments using R18D in stroke models.

## 2 Material and Methods

### 2.1 Peptides Used in Study

The R18D peptide, also known as ARG‐007 (H‐rrrrrrrrrrrrrrrrrr‐OH; lower case letters represent D‐arginine) was synthesized by Auspep (Australia; Batch #: GW461). R18D was purified by high‐performance liquid chromatography to at least 99% purity and subjected to peptide hydrolysis and amino acid liquid chromatography analysis to obtain a precise measure of peptide content (Auspep). For animal dosing, R18D was resuspended in saline (0.9% sodium chloride for injection; Baxter) to achieve the desired dose in a 600 μL volume.

### 2.2 Animals Ethics and Study Design

This study was performed following approval of an application submitted to the Committee for Ethical Conduct in the Care and Use of Laboratory Animals, confirming that the study complied with all relevant rules and regulations (Study #: IL‐2506‐271; Approval date: 15/06/2025). The study design was conducted in accordance with the Guide for the care and use of laboratory animals, STAIR [22] and ARRIVE [23] guidelines as appropriate.

### 2.3 Transient Middle Cerebral Artery Occlusion (MCAO) and Animal Dosing

MCAO studies were carried out by MD Biosciences (Isreal) under controlled laboratory conditions. Rats were subjected to 90 min of transient MCAO induced using an intraluminal filament as described previously [24], with modifications as outlined below. Briefly, rats underwent facemask anesthesia with 4% isoflurane (mix 30% oxygen/70% nitrous oxide) and maintenance with 1.5%–2% isoflurane. The animal's neck was then shaved, and a midline incision was made in the skin of the neck, and the tissue underneath was bluntly dissected. The right common carotid artery (CCA) and its junction with the external carotid artery (ECA) and internal carotid artery (ICA) were exposed by blunt dissection. The right CCA was then transiently closed by positioning around it a 3‐0 silk suture. The ECA was permanently occluded with the 3‐0 silk suture, and a vascular clip was placed on the ICA. A small hole was cut in the ECA and a silicon rubber‐coated monofilament (Doccol Corporation; Catalogue No: 403756PK10) was inserted into the ICA while avoiding entrance into the pterygopalatine artery. The filament was inserted ≈20 mm until a slight resistance was felt before a 3‐0 silk suture knot used to secure the filament. The left CCA was closed using a clip for the occlusion period. During the occlusion period, animals were removed from isoflurane and allowed to wake. During the surgical intervention, no heparin was used.

Fifty‐five minutes after occlusion, animals were assessed for stroke phenotype via the body swing/tail hang test [25]. All animals exhibited a stroke phenotype, and there were no exclusions prior to the dosing of animals. Ninety minutes after filament insertion (the beginning of MCAO), the animals were re‐anesthetized using isoflurane, the filament was removed, and the surgical incision was closed.

Sixty minutes after MCAO, treatments were administered intravenously in a 600 μL volume over 10 min through the right internal jugular vein using an infusion pump. Treatment groups consisted of the vehicle (0.9% sodium chloride) and three treatment arms receiving escalating doses of R18D of 325, 650, or 1100 nmol/kg. The doses were selected based on previous studies whereby R18D or its L‐enantiomer (R18) demonstrated neuroprotective actions in different rat stroke models [3, 4, 5, 6, 7]. Additionally, a pilot study was conducted to evaluate the effects of low‐dose R18D on infarct volume. In this study, treatment groups consisted of the vehicle and two R18D treatment arms at doses of 30 and 100 nmol/kg. In both studies, animals were randomly assigned to treatments, and all procedures were performed while being blinded to dosing status. To avoid hypothermia, rat cages were placed on a heating mat during the post‐surgical monitoring and housed in a holding room maintained at 26°C–28°C.

### 2.4 Animals Used and Sample Size

Male Sprague–Dawley rats weighing 290–320 g were obtained from Harlan Laboratories Israel Ltd. Animals were housed under controlled conditions on a 12‐h light–dark cycle, with free access to food and water prior to surgery for transient MCAO. For infarct volume analysis, the vehicle treatment group and R18D 325 nmol/kg treatment group each consisted of nine animals and the R18D 650 nmol/kg and 1100 nmol/kg treatment groups consisted of ten animals. Two animals died the day after surgery, one in the saline group (main study) and one in the R18D 325 nmol/kg group. The cause of death was not determined but was likely due to complications arising from the surgical procedure and/or the stroke.

### 2.5 Infarct Volume and Cerebral Hemisphere Swelling/Edema Measurement

Infarct volume was assessed 24 h after MCAO as previously described [24]. Briefly, 2 mm thick cerebral coronal brain slices were stained with pre‐warmed (37°C) 1% solution of 2,3,5 triphenyltetrazolium chloride (TTC, #T8877; Sigma‐Aldrich, USA) and digital images analyzed using ImageJ software (3rd edition, NIH, USA) by an operator blind to treatment status. The total infarct volume was determined by measuring the areas of infarcted tissue on both sides of each 2 mm slice. For statistical analysis, the higher infarct volume (mm^3^/% infarcted) of the 2 sides was used. The ratio of contralateral to stroke‐affected hemisphere areas was used to correct for cerebral oedema/hemisphere swelling. The cerebral oedema ratio was also used to determine percentage cerebral swelling of the stroke‐affected hemisphere.

### 2.6 Pharmacokinetic Analysis of R18D

For pharmacokinetic studies, blood from three rats subjected to MCAO from the different R18D treatment groups was collected 10, 15, 30 and 60 min and 2, 4 and 8 h after the start of the intravenous infusion. Blood was collected by a trained technician from the retro‐orbital sinus into an EDTA K3 tube and following centrifugation (3000 rpm/5 min), the plasma was transferred to a 1 mL screw top cryovial and stored at −80°C until shipping on dry ice to Agilex Biolabs (Australia).

Plasma concentrations were determined using a high‐performance liquid chromatography tandem mass spectrometry (HPLC/MS; Agilex Biolabs). Samples were analyzed alongside calibrators prepared in drug‐free matrix and concentration in the test samples was determined from back calculation from the standard curve. Briefly, analytes were separated by HPLC on an XBridge Premier BEH Amide column, and the eluates monitored by a Sciex 6500+ MS/MS detector in positive MRM mode (Sciex, Canada). The extract was then assayed against a calibration curve. The data are acquired and integrated by the data acquisition system Analyst (Sciex) linked directly to the Sciex 6500+ MS/MS and then processed in Watson LIMS (Thermo Scientific, USA), where applicable. The method range is from 30 ng/mL to 2400 ng/mL using 20 μL of rat plasma and has a run time of approximately 9 min per sample.

### 2.7 Statistical Analysis

Statistical analyses were performed using GraphPad Prism 11 (GraphPad Software, USA). Infarct volume, cerebral hemisphere swelling, and pharmacokinetic data are mean ± standard deviation of the mean (SD). Body weight measurements are mean ± standard error of the mean (SEM). For statistical analysis, treatment groups were compared to the vehicle group using Student's *t*‐test, and a value of *p* < 0.05 was considered to represent a significant difference.

## 3 Results

### 3.1 Infarct Volume and Cerebral Hemisphere Swelling Measurements

**FIGURE 1:**
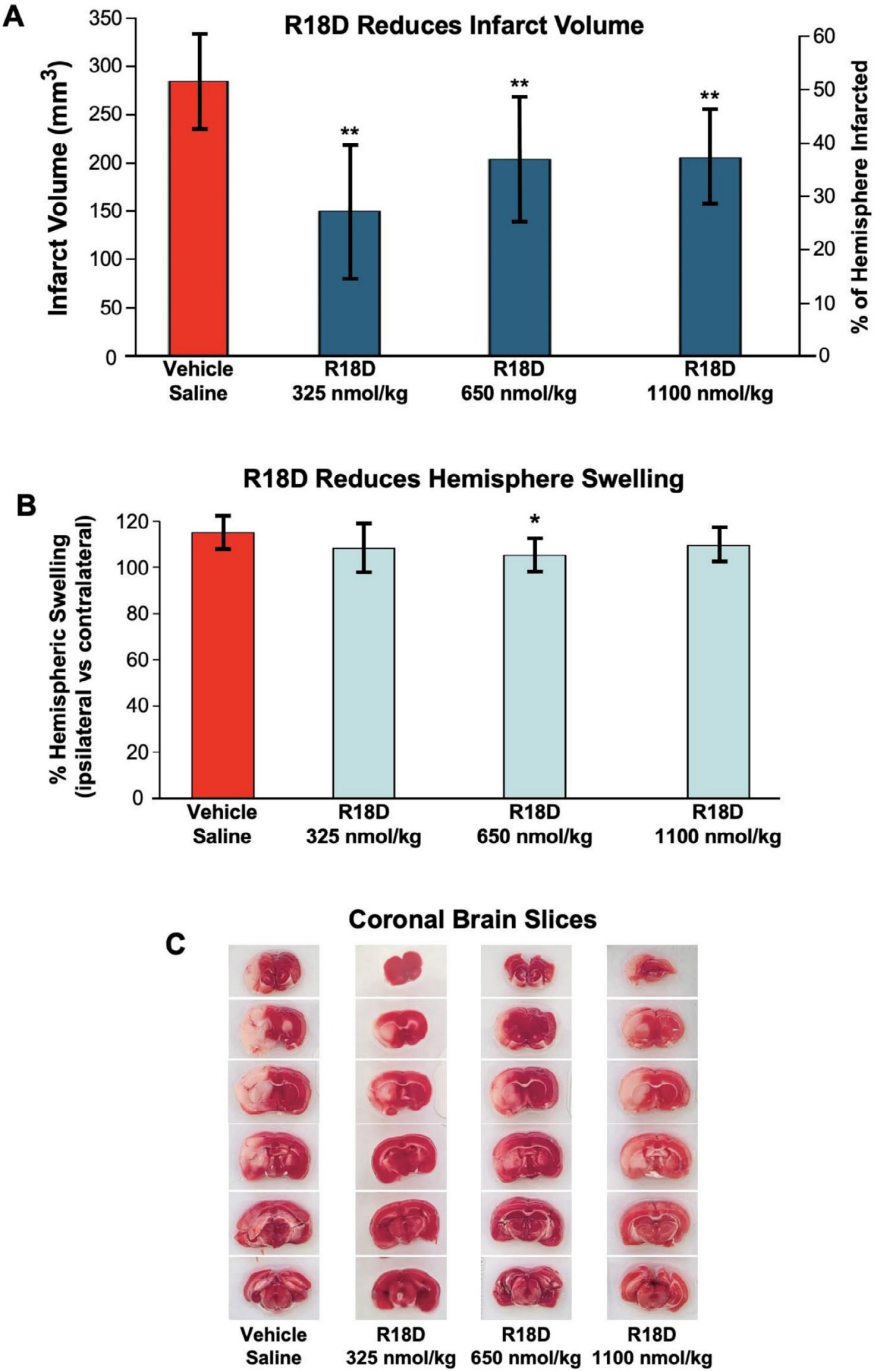
R18D neuroprotection following transient MCAO in the rat. (A) Infarct volume analysis for the different treatment groups as determined 24 h after transient MCAO (90 min). Vehicle (saline) or R18D treatments were administered intravenously 60 min after MCAO. Values are mean infarct volume/percentage hemisphere infarcted ± SD (*n* = 9–10). **p < 0.01 when compared to the vehicle control group. (B) Percentage cerebral hemisphere swelling for vehicle and R18D treatment groups. Values are means ± SD. *p < 0.05 when compared to the vehicle control group. (C) Representative images of TTC stained coronal brain slices from vehicle and R18D treatment groups.

Data on mean total infarct volume, percentage infarct volume reduction, and percentage cerebral hemisphere swelling as well as representative images of brain slices for each treatment group are presented in Table 1 and/or Figure 1A–C. These results show that the R18D significantly reduced infarct volume at doses of 325 nmol/kg by 47.2% (*p* = 0.0003), 650 nmol/kg by 28.4% (*p* = 0.008), and 1100 nmol/kg by 27.9% (*p* = 0.003; Figure 1A).

**TABLE 1:** Summary of infarct volume and cerebral hemisphere swelling in different treatment groups.

Treatment	Total infarct volume (mm^3^)^a^	Infarct size (% from the ipsilateral hemisphere)^a^	% Infarct volume reduction	% Hemisphere swelling	% Reduction in hemisphere swelling
Vehicle Saline	283.09 ± 50.28	51.74 ± 9.19	N/A	114.76 ± 7.36%	N/A
R18D 325 nmol/kg	149.4 ± 70.05**	27.30 ± 12.8**	47.2%	108.29 ± 10.53%	5.6%
R18D 650 nmol/kg	202.6 ± 64.94**	37.03 ± 11.87**	28.4%	105.26 ± 7.23%*	8.3%*
R18D 1100 nmol/kg	204.1 ± 48.26**	37.3 ± 8.82**	27.9%	109.56 ± 7.25%	4.5%

Abbreviation: N/A, not applicable.

^a^ Values are mean ± SD.

*p = < 0.05; **p = < 0.01 versus the vehicle group.

R18D significantly reduced cerebral hemisphere swelling at the 650 nmol/kg dose by 8.3% (*p* = 0.01), while the 325 nmol/kg and 1100 nmol/kg doses reduced swelling by 5.6% (*p* = 0.15) and 4.5% (*p* = 0.14), respectively (Figure 1B).

In the low‐dose pilot study, R18D did not produce a reduction in infarct volume or cerebral hemisphere swelling (Figure S1 and Table S1).

### 3.2 Body Weight Loss

While no significant differences were observed for body weight loss 1‐day post‐stroke, animals treated with R18D at the 325 nmol/kg dose recorded the lowest percentage weight loss: 7.35% versus 9.35% for the saline treated animals (Table 2).

**TABLE 2:** Summary of body weight measurements before and after MCAO.

Treatment/Study Day	Body weight (g)^a^, Day 0	Body weight (g), Day 1	Body weight % from baseline, Day 1	% Body weight loss from baseline, Day 1
Saline	300.0 ± 2.31	270.56 ± 2.99	90.65 ± 0.73	9.35
R18D 325 nmol/kg	303.3 ± 2.52	281.22 ± 3.07	92.65 ± 0.61	7.35
R18D 650 nmol/kg	308.6 ± 2.11	275.8 ± 3.33	89.38 ± 0.95	10.62
R18D 1100 nmol/kg	305.40 ± 2.57	274.9 ± 2.76	90.03 ± 0.72	9.97

^a^ Values are Mean ± SEM.

### 3.3 R18D Pharmacokinetic Data

**FIGURE 2:**
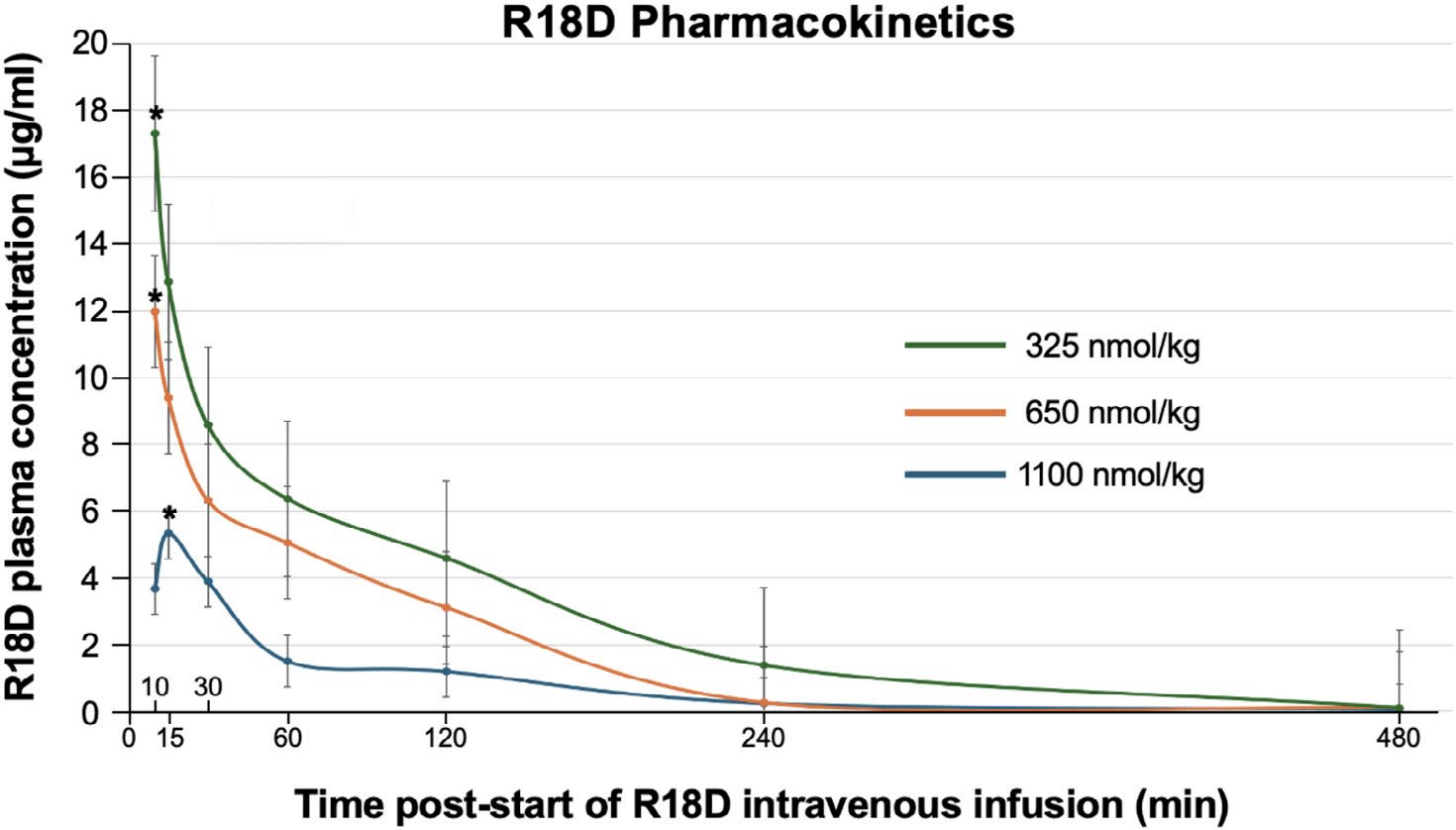
R18D pharmacokinetics following transient MCAO in the rat. Plasma R18D concentrations up to 8 h (480 min) after the start of the 10‐min intravenous infusion. Values are mean ± SD (*n* = 3). Time points on the *Y*‐axis indicate time blood was collected from rats from the start of the 10‐min intravenous R18D infusion. *Indicates *C*_max_.

Pharmacokinetic data for the R18D treatment groups are provided in Table 3 and Figure 2. R18D half‐life (72–78 min) and clearance (1.83–2.19 mL/min/kg) were similar for the three doses, whereas *C*_max_ (3670–31,500 ng/mL), *λ*_*z*_ (terminal rate constant; 0.53 to 0.58 1/h), and AUC values (e.g., AUC_last_ 6890 to 26,200 h*ng/mL) followed a typical dose response pattern.

**TABLE 3:** Summary of R18D pharmacokinetic data in rats undergoing MCAO.

	*T*_1/2_ (h/min)	AUC_last_ (h*ng/mL)	AUC_inf_ (h*ng/mL)	*C*_max_ ^a^ (ng/mL)	*λ*_*z*_ (1/h)	CL (mL/min/kg)
R18D 325 nmol/kg	1.3/78	6,890	7,000	5,328	0.53	2.19
R18D 650 nmol/kg	1.2/72	16,500	16,700	11,977	0.57	1.83
R18D 1100 nmol/kg	1.2/72	26,200	26,400	17,313	0.58	1.97

Abbreviations: AUC_inf_, Area under the concentration–time profile extrapolated to infinite time, calculated as AUC_last_ + *C*_last_/*λ*_*z*_, where *C*_last_ is the last quantifiable concentration; AUC_last_, Area under the plasma concentration–time curve from zero to the last quantifiable concentration; CL, Clearance; the volume of blood that is cleared of a drug per unit of time; *T*_½_, Terminal half‐life; *λ*_*z*_, Terminal rate constant (rate at which a drug is removed from the body), taken as the slope of the terminal phase of the log‐linear concentration–time profile.

^a^ *C*_max_: Maximum plasma concentration. In this study, *C*_max_ was observed 10 min after the start of IV infusion for the 650 and 1100 nmol/kg doses, and 15 min after the start of the infusion for the 325 nmol/kg dose and is considered representative of *T*_max_ (time to maximum plasma concentration).

## 4 Discussion

The present study has independently confirmed the neuroprotective effectiveness of R18D in a transient MCAO stroke model, demonstrating reductions in infarct volume by up to 47.2% and cerebral hemisphere swelling of 8.3% when administered 60 min after MCAO. These findings are consistent with previous experimental studies demonstrating the neuroprotective efficacy of R18D following permanent MCAO [6, 7], as well as functional improvement following ET‐1‐induced transient MCAO [8]. In addition, beneficial effects of the peptide have been reported in rat models of TBI [13, 14] and HIE [10, 11]. Collectively, these findings provide further strong evidence supporting the potential of R18D to reduce infarct volume and improve stroke outcomes when used as an adjunct to recanalization therapies in patients with acute ischemic stroke.

Unlike previous permanent MCAO stroke studies of R18D, which only examined a single dose (300 nmol/kg), the present study investigated a broader dose range (325, 650 and 1100 nmol/kg). Interestingly, the lowest R18D dose was found to be more effective than the higher doses, reducing infarct volume by nearly half, whereas the higher doses achieved reductions of only approximately one‐third. Furthermore, data from a pilot study using this model demonstrated that lower doses of R18D (30 and 100 nmol/kg) were not effective at reducing infarct volume (Figure S1).

Taken together, the dose–response data from this transient MCAO model indicate that the optimal effective dose of R18D lies between 100 and 650 nmol/kg, and that doses either lower or higher than 325 nmol/kg are unlikely to provide substantially greater reductions in infarct volume. These findings are consistent with our dose–response study of R18D (100, 300, 1000 nmol/kg) in a rat ET‐1 MCAO model, which assessed functional outcomes up to 56 days post‐stroke [8]. In the ET‐1 stroke study, the R18D dose of 100 nmol/kg provided little benefit, whereas the 300 and 1000 nmol/kg doses improved several functional outcomes.

Consistent with previous studies, R18D reduced cerebral hemisphere swelling, with the 650 nmol/kg dose producing a statistically significant reduction of 8.3%. Previous studies in our laboratory have demonstrated that both R18D and R18 can significantly reduce hemisphere swelling by 27%–42% following transient or permanent MCAO in rats [5, 6, 21]. Differences in stroke severity and/or strain‐ or colony‐specific variations in the response to ischemic injury may explain why the effect of R18D on hemisphere swelling was less pronounced in the present study compared with previous findings.

This study is the first to report the pharmacokinetic profile of R18D, and importantly in a post‐stroke setting that more closely reflects the clinical context. The half‐life of R18D was comparable across the three administered doses, ranging from 72 to 78 min. In a Phase 1 study in healthy volunteers, intravenous administration of R18D at a dose of 100 nmol/kg demonstrated a half‐life of 15.8 h (948 min; unpublished observation). Similarly, in a Phase 2 study in stroke patients treated with R18D at the same dose, the observed half‐life ranged from 12.1 to 14.4 h (unpublished observation).

By comparison, following intravenous administration, the similarly sized cationic arginine‐rich peptide, TAT‐NR2B9c (NA‐1; Nerinetide) exhibits a half‐life of only 4–10 min in rats, macaques and humans at doses ranging from 1000 and 3000 nmol/kg [26]. The extended half‐life for R18D is likely attributable to its D‐enantiomer structure, which confers resistance to proteolytic degradation [7, 17], as well as its high positive charge (+18), which may promote rapid binding to negatively charged serum proteins such as albumin and α1‐acid glycoprotein [27, 28]. Indeed, we have previously demonstrated that R18D remains stable following prolonged exposure (e.g., 20 h) to a trypsin‐like enzyme (TrypLE Express; Thermo Fischer Scientific), alteplase (tPA), tenecteplase (TNK), plasmin and culture supernatant derived neurons undergoing excitotoxic cell death [7, 17].

The enhanced metabolic stability and prolonged half‐life of R18D in humans are particularly advantageous in the acute stroke setting, as they may reduce the need for repeated dosing, provide sustained neuroprotection during both the early ischemic and the later reperfusion and neuroinflammatory phases of injury, and enable the use of lower effective doses, thereby potentially reducing the risk of adverse side effects.

As mentioned in the Introduction, CARPs such as R18D are likely to possess multimodal neuroprotective mechanisms of action in vivo, thereby providing the best opportunity of reducing the cascade of damaging processes involved in ischaemic brain injury following stroke. For example, R18D and other CARPs have been shown to reduce excitotoxic neuronal calcium influx [10, 16], exert beneficial effects on mitochondria [18, 29, 30, 31, 32], inhibit proteolytic enzymes [33, 34], down‐regulate cell surface TNF receptors [35], inhibit ion channels [36, 37, 38, 39, 40], scavenge free radicals, reduce lipid peroxidation and activate the critical antioxidant regulator nuclear factor erythroid 2‐related factor 2 (NRF2) [41, 42, 43, 44, 45]. In addition, CARPs have been reported to inhibit the activity of the proteasome [34, 46, 47], modulate immune and inflammatory responses [42, 48, 49, 50, 51, 52] and induce cell survival signaling pathways [53, 54].

Furthermore, the ability of R18D to reduce brain edema may be related to its capacity to indirectly inhibit activation of matrix metalloproteinases (MMP), thereby helping to maintain blood brain barrier integrity. For example, short‐chained poly‐arginine peptides and arginine‐rich cell‐penetrating peptides are potent inhibitors of proprotein convertases involved in the activation of matrix metalloproteinases [55, 56, 57, 58].

This study has several limitations. First, the efficacy of R18D in the MCAO stroke model was only assessed at a 24‐h endpoint and did not include functional assessment of the animals. Therefore, further studies may be required to confirm that the neuroprotection observed with R18D in this rat transient MCAO model translates into sustained histological benefit and long‐term functional recovery. However, previous studies in our laboratory using R18D and R18 in rodent and non‐human primate stroke models have demonstrated long‐lasting neuroprotection in terms of both tissue preservation and functional improvement [8, 9]. Second, only a single treatment time‐point was examined; therefore, the therapeutic time window for R18D in the model was not determined. While defining therapeutic time windows is valuable in experimental stroke models, in the clinical setting, stroke heterogeneity means that individualized “tissue windows” may provide a more accurate assessment of the potential to salvage vulnerable brain tissue. Finally, the study used young rats, despite stroke being more common in the elderly, often with multiple comorbidities. This may influence stroke severity, drug pharmacokinetics and adverse events, potentially limiting the translatability of these findings.

## 5 Conclusions

In conclusion, this independent study confirms that the R18D peptide exhibits a favorable pharmacokinetic profile and is capable of reducing infarct volume and cerebral oedema in a rat stroke model of transient occlusion followed by reperfusion. This demonstration of R18D's pharmacokinetics and efficacy in a reperfusion‐associated stroke model provides further evidence that the peptide has the potential to offer clinical benefit for stroke patients, both through direct neuroprotection and by extending the therapeutic time window for endovascular recanalization therapies.

## Author Contributions

B.P.M., S.K.G., M.G.T. and S.M. designed and supervised this research; A.W., L.F., L.F., D.K., G.M., A.S., and K.K.‐Z. conducted experiments; A.S., L.F. (Feinstein), D.K. and G.M. performed statistical analysis; B.P.M., S.K.G. and M.G.T. wrote and edited the manuscript; S.M. and A.S. verified the data. All authors have reviewed the final version of the manuscript and provided their approval for its submission.

## Funding

This research was funded by Argenica Therapeutics.

## Ethics Statement

All animal experiments were conducted in accordance with the Committee for Ethical Conduct in the Care and Use of Laboratory Animals (Study #: IL‐2506‐271; Approval date: 15/06/2025).

## Consent

The authors have nothing to report.

## Conflicts of Interest

B.P.M. is the holder of several patents regarding the use of cationic arginine‐rich peptides as neuroprotective treatments. In addition, B.P.M., is a shareholder of Argenica Therapeutics, which is a company developing R18D as a therapeutic for stroke and other neurological disorders. M.G.T. (Vice President, Clinical Development) and S.K.G. (Vice President, Product Development) are employed by Argenica Therapeutics. The other authors declare no conflicts of interest and Argenica Therapeutics had no role in the execution of the study.

## Supporting information


**Figure S1:** Pilot study; infarct volume analysis for vehicle (saline) and R18D treatment groups (30 and 100 nmol/kg) as determined 24 h after transient MCAO (90 min).

**Table S1:** Pilot study; summary of infarct volume and cerebral hemisphere swelling in different treatment groups.

## Data Availability

The data presented in this study are available on reasonable request from the corresponding author.

## References

[1] M. Glavan , J. Liu , G. Sampaio Silva , et al., “Endovascular Thrombectomy for Acute Stroke: Evolving Eligibility Criteria and Adjunct Therapies,” Lancet Neurology 25, no. 1 (2026): 61–76, 10.1016/S1474-4422(25)00356-4. 41389830

[2] B. P. Meloni , F. L. Mastaglia , and N. W. Knuckey , “Cationic Arginine‐Rich Peptides (CARPs): A Novel Class of Neuroprotective Agents With a Multimodal Mechanism of Action,” Frontiers in Neurology 11 (2020): 108. 32158425 10.3389/fneur.2020.00108 PMC7052017

[3] D. Milani , V. W. Clark , J. L. Cross , R. S. Anderton , N. W. Knuckey , and B. P. Meloni , “Poly‐Arginine Peptides Reduce Infarct Volume in a Permanent Middle Cerebral Artery Rat Stroke Model,” BMC Neuroscience 17 (2016): 19, 10.1186/s12868-016-0253-z. 27142074 PMC4855717

[4] D. Milani , N. W. Knuckey , J. L. Cross , R. S. Anderton , and B. P. Meloni , “The R18 Poly‐Arginine Peptide Is More Effective Than the TAT‐NR2B9c (NA‐1) Peptide When Administered 60 Minutes After Permanent Middle Cerebral Artery Occlusion in the Rat,” Stroke Research and Treatment 2016 (2016): 2372710, 10.1155/2016/2372710. 27247825 PMC4877491

[5] D. Milani , J. L. Cross , R. S. Anderton , et al., “Neuroprotective Efficacy of R18 Poly‐Arginine and NA‐1 (TAT‐NR2B9c) Peptides Following Transient Middle Cerebral Artery Occlusion in the Rat,” Neuroscience Research 114 (2017): 9–15, 10.1016/j.neures.2016.11.001. 27639457

[6] D. Milani , M. C. Bakeberg , J. L. Cross , et al., “Comparison of Neuroprotective Efficacy of Poly‐Arginine R18 and R18D (D‐Enantiomer) Peptides Following Permanent Middle Cerebral Artery Occlusion in the Wistar Rat and In Vitro Toxicity Studies,” PLoS One 13 (2018): e0193884, 10.1371/journal.pone.0193884. 29513757 PMC5841795

[7] D. Milani , V. W. Clark , K. W. Feindel , et al., “Comparative Assessment of the Proteolytic Stability and Impact of Poly‐Arginine Peptides R18 and R18D on Infarct Growth and Penumbral Tissue Preservation Following Middle Cerebral Artery Occlusion in the Sprague Dawley Rat,” Neurochemical Research 46, no. 5 (2021): 1166–1176, 10.1007/s11064-020-03216-7. 33523394

[8] B. P. Meloni , S. M. South , D. A. Gill , et al., “Poly‐Arginine Peptides R18 and R18D Improve Functional Outcomes After Endothelin‐1 (ET‐1)‐Induced Stroke in the Sprague Dawley Rat,” Journal of Neuropathology and Experimental Neurology 78, no. 5 (2019): 426–435, 10.1093/jnen/nlz027. 30888409

[9] B. P. Meloni , Y. Chen , K. A. Harrison , et al., “Poly‐Arginine Peptide‐18 (R18) Reduces Brain Injury and Improves Functional Outcomes in a Non‐Human Primate Stroke Model,” Neurotherapeutics 17 (2020): 627–634, 10.1007/s13311-019-00809-1. 31833045 PMC7283416

[10] A. B. Edwards , J. L. Cross , R. S. Anderton , et al., “Poly‐Arginine R18 and R18D (D‐Enantiomer) Peptides Reduce Infarct Volume and Improve Behavioural Outcomes Following Perinatal Hypoxic‐Ischaemic Encephalopathy in the P7 Rat,” Molecular Brain 11 (2018): 8, 10.1186/s13041-018-0355-1. 29426351 PMC5810179

[11] A. B. Edwards , R. S. Anderton , N. W. Knuckey , and B. P. Meloni , “Assessment of Therapeutic Window for Poly‐Arginine‐18D (R18D) in a P7 Rat Model of Perinatal Hypoxic‐Ischaemic Encephalopathy,” Journal of Neuroscience Research 96 (2018): 1816–1826, 10.1002/jnr.24315. 30146697

[12] L. S. Chiu , R. S. Anderton , J. L. Cross , et al., “Assessment of Neuroprotective Peptides Poly‐Arginine R18, COG1410, and APP96‐110 in Experimental Traumatic Brain Injury and In Vitro Excitotoxicity,” Translational Neuroscience 8 (2017): 147–157, 10.1515/tnsci-2017-0021. 29177102 PMC5700203

[13] L. S. Chiu , R. S. Anderton , J. L. Cross , V. W. Clark , N. W. Knuckey , and B. P. Meloni , “Poly‐Arginine Peptide R18D Reduces Neuroinflammation and Functional Deficits Following Traumatic Brain Injury in the Long Evans Rat,” International Journal of Peptide Research and Therapeutics 16 (2019): 1–8, 10.1007/s10989-018-09799-8.

[14] L. S. Chiu , R. S. Anderton , V. W. Clark , J. L. Cross , N. W. Knuckey , and B. P. Meloni , “Effect of Poly‐Arginine Peptide R18D Following a Traumatic Brain Injury in Sprague‐Dawley Rats,” Current Therapeutic Research 92 (2020): 100584, 10.1016/j.curtheres.2020.100584. 32322314 PMC7163064

[15] H. Batulu , G. J. Du , D. Z. Li , D. Sailike , Y. H. Fan , and D. Geng , “Effect of Poly‐Arginine R18 on Neurocyte Cell Growth via Autophagy in Traumatic Brain Injury,” Experimental and Therapeutic Medicine 17, no. 5 (2019): 4109–4115, 10.3892/etm.2019.7423. 30988787 PMC6447892

[16] B. P. Meloni , L. M. Brookes , V. W. Clark , et al., “Poly‐Arginine and Arginine‐Rich Peptides Are Neuroprotective in Stroke Models,” Journal of Cerebral Blood Flow and Metabolism 35 (2015): 993–1004, 10.1038/jcbfm.2015.11. 25669902 PMC4640246

[17] B. P. Meloni , D. J. Blacker , A. B. Edwards , and N. W. Knuckey , “Impact of Poly‐Arginine Peptides R18D and R18 on Alteplase and Tenecteplase Thrombolysis In Vitro, and Neuroprotective Stability to Proteolysis,” Journal of Thrombosis and Thrombolysis 53 (2022): 1–13. 35305237 10.1007/s11239-022-02642-4 PMC9259545

[18] G. MacDougall , R. S. Anderton , A. Trimble , F. L. Mastaglia , N. W. Knuckey , and B. P. Meloni , “Polyarginine‐18 (R18) Confers Neuroprotection Against Excitotoxicity Through Glutamate Receptor Modulation, Intracellular Calcium Reduction, and Preservation of Mitochondrial Function,” Molecules 25, no. 13 (2020): 2977, 10.3390/molecules25132977. 32610439 PMC7412265

[19] J. E. Kenna , R. S. Anderton , N. W. Knuckey , and B. P. Meloni , “Assessment of Recombinant Tissue Plasminogen Activator (rtPA) Toxicity in Cultured Neural Cells and Subsequent Treatment With Poly‐Arginine Peptide R18D,” Neurochemical Research 45 (2020): 1215–1229, 10.1007/s11064-020-03004-3. 32140956

[20] L. Liddle , R. Reinders , S. M. South , et al., “Poly‐Arginine‐18 Peptides Do Not Exacerbate Bleeding, or Improve Functional Outcomes Following Collagenase‐Induced Intracerebral Hemorrhage in the Rat,” PLoS One 14 (2019): e0224870, 10.1371/journal.pone.0224870. 31697775 PMC6837498

[21] B. P. Meloni , D. Milani , J. L. Cross , et al., “Assessment of the Neuroprotective Effects of Arginine‐Rich Protamine Peptides, Poly‐Arginine Peptides (R12‐Cyclic, R22) and Arginine‐Tryptophan Containing Peptides Following In Vitro Excitotoxicity and/or Permanent Middle Cerebral Artery Occlusion in Rats,” Neuromolecular Medicine 19 (2017): 271–285, 10.1007/s12017-017-8441-x. 28523591

[22] M. Fisher , G. Feuerstein , D. W. Howells , et al., “Update of the Stroke Therapy Academic Industry Roundtable Preclinical Recommendations,” Stroke 40, no. 6 (2009): 2244–2250, 10.1161/STROKEAHA.108.541128. 19246690 PMC2888275

[23] C. Kilkenny , W. J. Browne , I. C. Cuthill , M. Emerson , and D. G. Altman , “Improving Bioscience Research Reporting: The ARRIVE Guidelines for Reporting Animal Research,” Journal of Pharmacology and Pharmacotherapeutics 1, no. 2 (2010): 94–99, 10.4103/0976-500X.72351. 21350617 PMC3043335

[24] K. Campbell , N. W. Knuckey , L. M. Brookes , and B. P. Meloni , “Efficacy of Mild Hypothermia (35°C) and Moderate Hypothermia (33°C) With and Without Magnesium When Administered 30 Min Post‐Reperfusion After 90 Min of Middle Cerebral Artery Occlusion in Spontaneously Hypertensive Rats,” Brain Research 1502 (2013): 47–54, 10.1016/j.brainres.2013.01.041. 23370002

[25] E. Ingberg , J. Gudjonsdottir , E. Theodorsson , A. Theodorsson , and J. O. Ström , “Elevated Body Swing Test After Focal Cerebral Ischemia in Rodents: Methodological Considerations,” BMC Neuroscience 16 (2015): 50, 10.1186/s12868-015-0189-8. 26242584 PMC4525734

[26] D. Mayor‐Nunez , Z. Ji , X. Sun , L. Teves , J. D. Garman , and M. Tymianski , “Plasmin‐Resistant PSD‐95 Inhibitors Resolve Effect‐Modifying Drug–Drug Interactions Between Alteplase and Nerinetide in Acute Stroke,” Science Translational Medicine 13, no. 588 (2021): eabb1498, 10.1126/scitranslmed.abb1498. 33827973

[27] L. T. Nguyen , J. K. Chau , N. A. Perry , L. de Boer , S. A. J. Zaat , and H. J. Vogel , “Serum Stabilities of Short Tryptophan‐ and Arginine‐Rich Antimicrobial Peptide Analogs,” PLoS One 5, no. 9 (2010): e12684, 10.1371/journal.pone.0012684. 20844765 PMC2937036

[28] E. Schartmann , S. Schemmert , N. Niemietz , et al., “In Vitro Potency and Preclinical Pharmacokinetic Comparison of All‐D‐Enantiomeric Peptides Developed for the Treatment of Alzheimer's Disease,” Journal of Alzheimer's Disease 64, no. 3 (2018): 859–873, 10.3233/JAD-180165. PMC6218115 29966196

[29] M. P. Rigobello , E. Barzon , O. Marin , et al., “Effect of Polycation Peptides on Mitochondrial Permeability Transition,” Biochemical and Biophysical Research Communications 217 (1995): 144–149, 10.1006/bbrc.1995.2412. 8526902

[30] K. Zhao , G. M. Zhao , D. Wu , et al., “Cell‐Permeable Peptide Antioxidants Targeted to Inner Mitochondrial Membrane Inhibit Mitochondrial Swelling, Oxidative Cell Death, and Reperfusion Injury,” Journal of Biological Chemistry 279 (2004): 34682–34690, 10.1074/jbc.M405053200. 15178689

[31] J. Marshall , K. Y. Wong , C. N. Rupasinghe , et al., “Inhibition of N‐Methyl‐D‐Aspartate‐Induced Retinal Neuronal Death by Polyarginine Peptides Is Linked to the Attenuation of Stress‐Induced Hyperpolarization of the Inner Mitochondrial Membrane Potential,” Journal of Biological Chemistry 290 (2015): 22030–22048, 10.1074/jbc.M115.662083. 26100636 PMC4571956

[32] A. V. Birk , W. M. Chao , S. Liu , Y. Soong , and H. H. Szeto , “Disruption of Cytochrome c Heme Coordination Is Responsible for Mitochondrial Injury During Ischemia,” Biochimica et Biophysica Acta 1847 (2015): 1075–1084, 10.1016/j.bbabio.2015.06.006. 26071084 PMC4547887

[33] M. Horn , M. Pavlík , L. Dolecková , M. Baudys , and M. Mares , “Arginine‐Based Structures Are Specific Inhibitors of Cathepsin C. Application of Peptide Combinatorial Libraries,” European Journal of Biochemistry 267, no. 11 (2000): 3330–3336, 10.1046/j.1432-1327.2000.01364.x. 10824120

[34] A. Kloss , P. Henklein , D. Siele , et al., “The Cell‐Penetrating Peptide Octa‐Arginine Is a Potent Inhibitor of Proteasome Activities,” European Journal of Pharmaceutics and Biopharmaceutics 72 (2009): 219–225, 10.1016/j.ejpb.2008.10.004. 19027853

[35] M. Fotin‐Mleczek , S. Welte , O. Mader , et al., “Cationic Cell‐Penetrating Peptides Interfere With TNF Signalling by Induction of TNF Receptor Internalization,” Journal of Cell Science 118 (2005): 3339–3351, 10.1242/jcs.02489. 16079278

[36] A. V. Ferrer‐Montiel , J. M. Merino , S. E. Blondelle , E. Perez‐Payà , R. A. Houghten , and M. Montal , “Selected Peptides Targeted to the NMDA Receptor Channel Protect Neurons From Excitotoxic Death,” Nature Biotechnology 16 (1998): 286–291, 10.1038/nbt0598-286. 9528011

[37] T. Brustovetsky , J. J. Pellman , X. F. Yang , R. Khanna , and N. Brustovetsky , “Collapsin Response Mediator Protein 2 (CRMP2) Interacts With N‐Methyl‐D‐Aspartate (NMDA) Receptor and Na+/Ca2+ Exchanger and Regulates Their Functional Activity,” Journal of Biological Chemistry 289 (2014): 7470–7482, 10.1074/jbc.M113.518472. 24474686 PMC3953261

[38] R. Planells‐Cases , A. Aracil , J. M. Merino , et al., “Arginine‐Rich Peptides Are Blockers of VR‐1 Channels With Analgesic Activity,” FEBS Letters 481 (2000): 131–136, 10.1016/S0014-5793(00)01969-3. 10996311

[39] A. García‐Caballero , V. M. Gadotti , P. Stemkowski , et al., “The Deubiquitinating Enzyme USP5 Modulates Neuropathic and Inflammatory Pain by Enhancing Cav3.2 Channel Activity,” Neuron 83 (2014): 1144–1158, 10.1016/j.neuron.2014.07.041. 25189210

[40] D. S. Lebedev , E. V. Kryukova , I. A. Ivanov , et al., “Oligoarginine Peptides, a New Family of Nicotinic Acetylcholine Receptor Inhibitors,” Molecular Pharmacology 96, no. 5 (2019): 664–673, 10.1124/mol.119.117713. 31492697

[41] M. H. Kown , M. A. Lijkwan , C. L. Jahncke , et al., “L‐Arginine Polymers Enhance Coronary Flow and Reduce Oxidative Stress Following Cardiac Transplantation in Rats,” Journal of Thoracic and Cardiovascular Surgery 126 (2003): 1065–1070, 10.1016/S0022-5223(03)00470-0. 14566248

[42] C. Aluganti Narasimhulu , K. Selvarajan , M. Brown , and S. Parthasarathy , “Cationic Peptides Neutralize Ox‐LDL, Prevent Its Uptake by Macrophages, and Attenuate Inflammatory Response,” Atherosclerosis 236 (2014): 133–141, 10.1016/j.atherosclerosis.2014.06.020. 25036240

[43] S. M. Mandal , R. Bharti , W. F. Porto , et al., “Identification of Multifunctional Peptides From Human Milk,” Peptides 56 (2014): 84–93, 10.1016/j.peptides.2014.03.020. 24703967

[44] J. Tu , X. Zhang , Y. Zhu , et al., “Cell‐Permeable Peptide Targeting the Nrf2‐Keap1 Interaction: A Potential Novel Therapy for Global Cerebral Ischemia,” Journal of Neuroscience 35, no. 44 (2015): 14727–14739, 10.1523/JNEUROSCI.1304-15.2015. 26538645 PMC4635127

[45] B. B. Saikia , S. Alikunju , Y. A. Poovanthodi , Z. Kassim , and P. M. A. Muneer , “Nrf2 Activator Peptide Protects the Brain From Cerebral Vascular Dysfunction in Alcohol Ingestion,” JCI Insight 11 (2026): e188004, 10.1172/jci.insight.188004. 41701913 PMC13043087

[46] M. Gaczynska , P. A. Osmulski , Y. Gao , et al., “Proline‐ and Arginine‐Rich Peptides Constitute a Novel Class of Allosteric Inhibitors of Proteasome Activity,” Biochemistry 42 (2003): 8663–8670, 10.1021/bi0341653. 12873125

[47] A. Anbanandam , D. C. Albarado , D. C. Tirziu , M. Simons , and S. Veeraraghavan , “Molecular Basis for Proline‐ and Arginine‐Rich Peptide Inhibition of Proteasome,” Journal of Molecular Biology 384 (2008): 219–227, 10.1016/j.jmb.2008.09.021. 18823992 PMC2632303

[48] D. T. Laskowitz , A. D. Thekdi , S. D. Thekdi , et al., “Downregulation of Microglial Activation by Apolipoprotein E and apoE‐Mimetic Peptides,” Experimental Neurology 167 (2001): 74–85, 10.1006/exnr.2000.7545. 11161595

[49] A. L. Hilchie , K. Wuerth , and R. E. Hancock , “Immune Modulation by Multifaceted Cationic Host Defense (Antimicrobial) Peptides,” Nature Chemical Biology 9 (2013): 761–768, 10.1038/nchembio.1367. 24231617

[50] L. H. Li , T. C. Ju , C. Y. Hsieh , et al., “A Synthetic Cationic Antimicrobial Peptide Inhibits Inflammatory Response and the NLRP3 Inflammasome by Neutralizing LPS and ATP,” PLoS One 12 (2017): e0182057, 10.1371/journal.pone.0182057. 28750089 PMC5531531

[51] G. Datta , M. Chaddha , S. P. Handattu , et al., “ApoE Mimetic Peptide Reduces Plasma Lipid Hydroperoxide Content With a Concomitant Increase in HDL Paraoxonase Activity,” Advances in Experimental Medicine and Biology 660 (2010): 1–4, 10.1007/978-1-4419-1212-3_1. 20221865

[52] Q. Zhang , Y. Jiang , X. He , L. Liu , and X. Zhang , “Study of an Arginine‐ and Tryptophan‐Rich Antimicrobial Peptide in Peri‐Implantitis,” Frontiers in Bioengineering and Biotechnology 12 (2025): 1486213, 10.3389/fbioe.2024.1486213. 39840136 PMC11747041

[53] Q. Gu , L. Zhai , X. Feng , et al., “Apelin‐36, a Potent Peptide, Protects Against Ischemic Brain Injury by Activating the PI3K/Akt Pathway,” Neurochemistry International 63 (2013): 535–540, 10.1016/j.neuint.2013.06.004. 24083989

[54] Y. Yang , X. J. Zhang , L. T. Li , et al., “Apelin‐13 Protects Against Apoptosis by Activating AMP‐Activated Protein Kinase Pathway in Ischemic Stroke,” Peptides 75 (2016): 96–100, 10.1016/j.peptides.2015.11.013. 26631263

[55] A. Cameron , J. Appel , R. A. Houghten , and I. Lindberg , “Polyarginines Are Potent Furin Inhibitors,” Journal of Biological Chemistry 275 (2000): 36741–36749, 10.1074/jbc.M003848200. 10958789

[56] M. M. Kacprzak , J. R. Peinado , M. E. Than , et al., “Inhibition of Furin by Polyarginine‐Containing Peptides: Nanomolar Inhibition by Nona‐D‐Arginine,” Journal of Biological Chemistry 279 (2004): 36788–36794, 10.1074/jbc.M406192200. 15197180

[57] M. Fugere , J. Appel , R. A. Houghten , et al., “Short Polybasic Peptide Sequences Are Potent Inhibitors of PC5/6 and PC7,” Molecular Pharmacology 71 (2007): 323–332, 10.1124/mol.106.031497. 17012622

[58] B. Ramos‐Molina , A. N. Lick , A. Nasrolahi Shirazi , et al., “Cationic Cell‐Penetrating Peptides Are Potent Furin Inhibitors,” PLoS One 10, no. 6 (2015): e0130417, 10.1371/journal.pone.0130417. 26110264 PMC4482483

