# Supplementary material for: Pharmacokinetics and Neuroprotective Efficacy of Poly‐Arginine Peptide R18D in Sprague Dawley Rats Subjected to Transient Intraluminal Filament Middle Cerebral Artery Occlusion Stroke
Source: CNS Neurosci Ther. 2026 Jun 3;32(6):e70969. doi: 10.1002/cns.70969 (PMC13239245; doi:10.1002/cns.70969)
Supplement: Supplementary file 1 — Figure S1: Pilot study; infarct volume analysis for vehicle (saline) and R18D treatment groups (30 and 100 nmol/kg) as determined 24 h after transient MCAO (90 min). [file CNS-32-e70969-s001.pptx]

## Slide 1
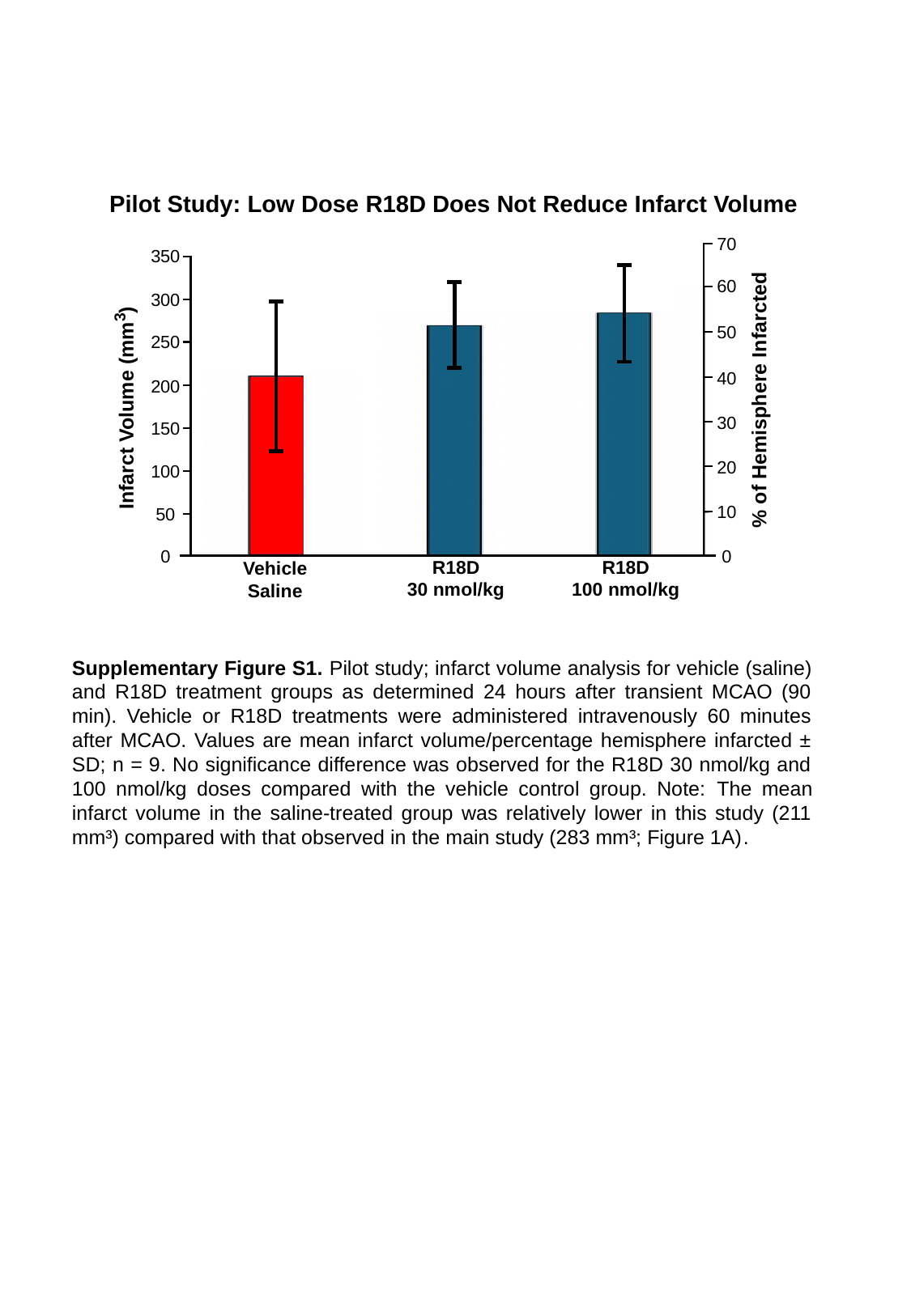

Pilot Study: Low Dose R18D Does Not Reduce Infarct Volume
70
350
60
300
50
250
40
200
% of Hemisphere Infarcted
Infarct Volume (mm3)
30
150
20
100
10
50
0
0
R18D
30 nmol/kg
R18D
100 nmol/kg
Vehicle
Saline
Supplementary Figure S1. Pilot study; infarct volume analysis for vehicle (saline) and R18D treatment groups as determined 24 hours after transient MCAO (90 min). Vehicle or R18D treatments were administered intravenously 60 minutes after MCAO. Values are mean infarct volume/percentage hemisphere infarcted ± SD; n = 9. No significance difference was observed for the R18D 30 nmol/kg and 100 nmol/kg doses compared with the vehicle control group. Note: The mean infarct volume in the saline-treated group was relatively lower in this study (211 mm³) compared with that observed in the main study (283 mm³; Figure 1A).
