# Supplementary material for: Pharmacokinetics and Neuroprotective Efficacy of Poly‐Arginine Peptide R18D in Sprague Dawley Rats Subjected to Transient Intraluminal Filament Middle Cerebral Artery Occlusion Stroke
Source: CNS Neurosci Ther. 2026 Jun 3;32(6):e70969. doi: 10.1002/cns.70969 (PMC13239245; doi:10.1002/cns.70969)
Supplement: Supplementary file 2 — Table S1: Pilot study; summary of infarct volume and cerebral hemisphere swelling in different treatment groups. [file CNS-32-e70969-s002.docx]

**Supplementary Table S1.** Pilot Study: Summary of infarct volume and cerebral hemisphere swelling in different treatment groups.

| **Treatment** | **Total infarct volume (mm^3^)^1^** | **Infarct size**  **(% from the ipsilateral hemisphere)^1^** | **% Hemisphere swelling** |
| --- | --- | --- | --- |
| **Vehicle**  **Saline** | 211.33 ± 35.57 | 40.48 ± 5.85 | 108.25 ± 3.34 |
| **R18D**  **30 nmol/kg** | 264.78 ± 22.13 | 51.84 ± 3.39 | 122.06 ± 5.60 |
| **R18D**  **100 nmol/kg** | 295.49 ± 30.27 | 54.62 ± 3.83 | 116.05 ± 4.44 |

^1^Vales are Mean ± SEM; N = 10.
